# Salvage of Cardiac Implantable Electronic Device Pocket Infection with Skin Erosion in Frail 92-Year-Old

**DOI:** 10.3390/jcdd9030081

**Published:** 2022-03-10

**Authors:** Marzia Giaccardi, Benito Baldauf, Ernest W. Lau, Stefan Borov, Hendrik Bonnemeier

**Affiliations:** 1 Department of Cardiology, Santa Maria Annunziata Hospital, Ponte a Niccheri, 50012 Florence, Italy; marzia.giaccardi@uslcentro.toscana.it; 2 Medical Faculty, Christian-Albrechts University Kiel, 24118 Kiel, Germany; stborov@gmail.com (S.B.); bonnemeier@t-online.de (H.B.); 3 Department of Cardiology, Royal Victoria Hospital, Grosvenor Road, Belfast BT12 6BA, UK; ernest.lau@btinternet.com; 4 Department of Cardiology, Krankenhaus Landshut-Achdorf, 84036 Landshut, Germany

**Keywords:** cardiac implantable electronic device, surgical site infection, revision, taurolidine

## Abstract

We reported the novel use of a taurolidine-containing antimicrobial solution in the successful salvage of a partially exposed and polymicrobially infected cardiac implantable electronic device pulse generator in a frail patient unfit for lead extraction. The old, salvaged device was entirely internalized, and there were no signs of recurrent infection at 9 months follow-up.

## 1. Introduction

The incidence of cardiac implantable electronic device (CIED) infection has risen faster than the volume of CIED surgical procedures over the past decades [1]. The reasons for this excessive rise are multiple and include a higher portion of older patients with more comorbidities among the new recipients due to expanded indications for CIED therapy, an increased percentage of device revisions (pulse generator replacement, with or without lead insertion and/or removal) compared to new implants due to the prolonged survival of the previous CIED recipients, and newer CIED models with more complex geometrical shapes to accommodate additional functionalities [2]. For CIED infection, whether the infection is confined locally to the pocket (i.e., abscess or skin erosion with exposure of hardware but negative blood culture) or extended systemically into the bloodstream (i.e., septicaemia, with or without detectable lead related endocarditis), the current guidelines recommend removal of all indwelling hardware [3,4]. Failure of complete system removal is associated with high rates of relapse of infection [5,6]. Lead removal, by either percutaneous lead extraction or open chest surgery, requires special equipment and expertise and may not be readily available and could even be infeasible for some patients. Moreover, percutaneous lead extraction is associated with a small risk of potentially fatal complications (e.g., cardiac avulsion, vascular avulsion, haemothorax, and cardiac tamponade) [7]. For these reasons, the performance of such procedures should be limited to centres with the necessary equipment and expertise [5]. However, alternative management options might be necessary in certain situations. Successful salvage of infected CIED systems with in situ sterilisation of the contaminated hardware has been reported [8,9]. We herein report the successful salvage of an exposed CIED pulse generator which had eroded through the skin and became contaminated with multiple microbes in a frail patient with taurolidine.

TauroPace™ (TauroPharm GmbH, Waldbüttelbrunn, Bavaria, Germany) is an antimicrobial agent specifically developed for use during CIED procedures. When dissolved in an aqueous solution (which contains povidone for the stability of taurolidine, the main ingredient [10]), taurolidine and cyclo-taurolidine are hydrolysed to the active compounds N-methylol-taurultam, taurultam, and N-methylol-taurinamide [11,12]. The metabolites degrade further through hydrolysis into taurine, a naturally occurring amino-sulfonic acid found in many biological tissues and secretions. During this process, 3 N-methylol groups are generated and these are believed to be the chemical active species responsible for taurolidine’s antimicrobial actions, which include denaturation of the pathogen cell wall and its surface virulence factors, inhibition of adherence to surfaces, interference with biofilm formation, and neutralisation of endotoxins and exotoxins [13,14,15,16,17]. After all the antimicrobial actions have been completed, the end-product of taurolidine metabolism, taurine, has antioxidant and anti-inflammatory properties and has been shown to enhance wound healing in vitro and in animal models [18,19,20].

## 2. Patient

**Figure 1:**
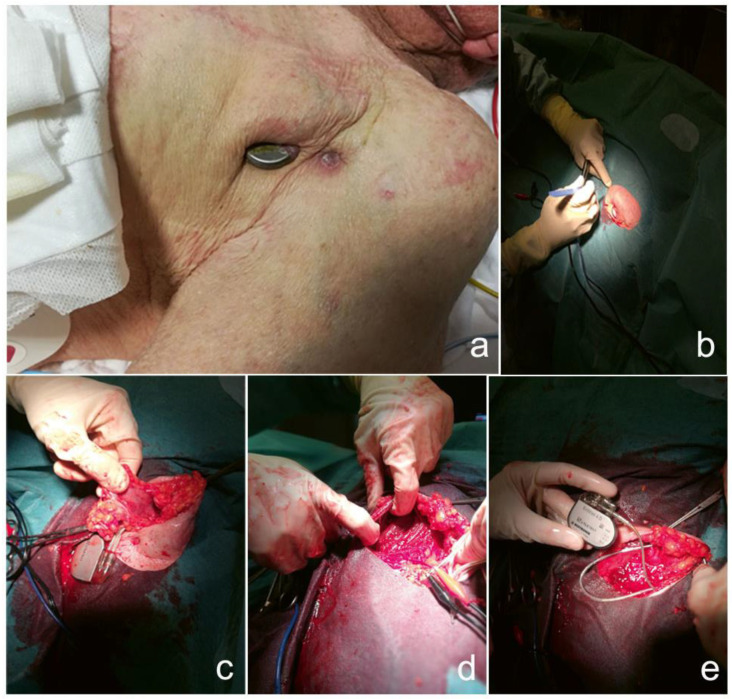
(**a**) Pulse generator protrusion through skin erosion (biofilm formation at the skin edges). (**b**) Initial elliptical incision around skin defect during salvage procedure. (**c**) Fibrous and subcutaneous tissues encasing the pulse generator and leads were only exposed after removal outside the patient’s body. (**d**) A new sub-pectoral pocket was fashioned. (**e**) The old pulse generator was re-attached to the old leads after ex vivo and in vivo sterilisation with TauroPace™ prior to insertion into the new sub-pectoral pocket.

In 2007, at the age of 77, a male patient was fitted with a dual chamber pacemaker for high degree atrio-ventricular heart block resulting in syncope. In February 2012, at the age of 82, he underwent his first pacemaker pulse generator replacement without any complications. In October 2020, at the age of 91, he underwent his second pacemaker pulse generator replacement. Eight months later, in June 2021, at the age of 92, the patient presented to the pacemaker department of the Santa Maria Annunziata Hospital (Florence, Italy) with pacemaker pulse generator protrusion through the skin. On inspection, about a quarter of the pulse generator was exposed outside the body (Figure 1a). The patient was referred to the electrophysiology service.

Swabs around the edges of the skin wound and the pocket revealed polymicrobial contamination with *Streptococcus agalactiae* and *Staphylococcus capitis*. The initial empirical antibiotic regime was adjusted according to the sensitivities from bacterial cultures and the patient’s history of allergies. The patient was systemically well. Blood cultures were negative. Echocardiography did not show any lead-related endocarditis. On these bases, bacterial infection appeared to be confined to the device pocket. By that time, the patient’s leads had been in situ for 15 years and he was frail with significant cognitive impairment. Taking all these factors into consideration, in deviation from the guidelines [3], the patient’s medical team proposed an attempt at salvaging the infected pacemaker in the first instance before consideration of more invasive higher risk lead extraction [6].The patient’s daughter, who was his legal guardian, gave consent to the management plan on his behalf.

**Figure 2:**
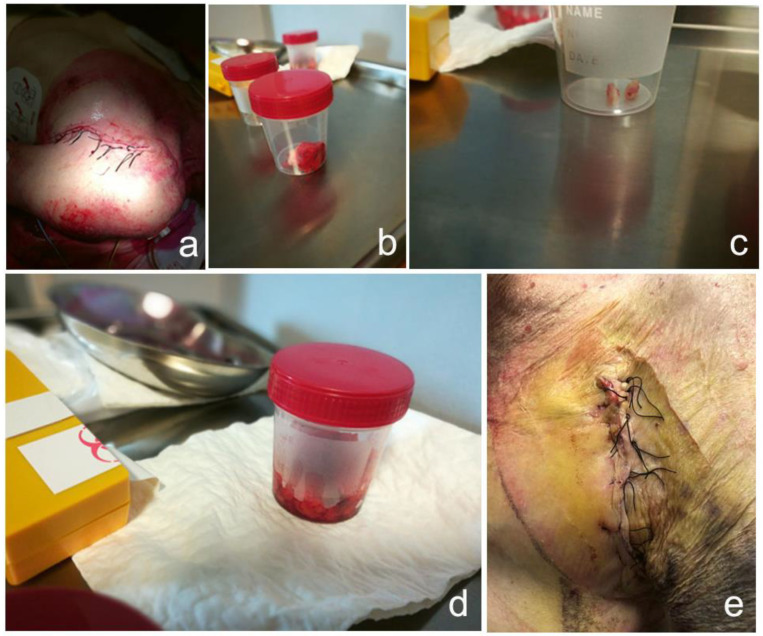
(**a**) The skin edges were closed with interrupted sutures. (**b**) Skin removed and sent in for histopathology. (**c**) Suture fixation sleeves sent in for microbiology. (**d**) Fibrous lining surrounding pulse generator and leads sent in for histopathology. (**e**) The pocket and wound looked healthy with no sign of recurrent infection, underlying abscess formation, or skin erosion 7 days post procedure.

During the pacemaker salvage procedure, an elliptical incision was made around the skin defect through which the pulse generator protruded (Figure 1b). The elliptical incision was then extended down to the surface of the pectoralis major to deliver the pulse generator and the attached leads (still encased in subcutaneous tissues) en bloc free from the pocket. The adherent (and infectious) fibrous and subcutaneous tissues (Figure 1c) surrounding the pulse generator and the leads were dissected and removed (Figure 2b,d). The pulse generator was detached from the leads, wrapped in a swab, and placed in a gallipot containing TauroPace™. All sutures and the anchor sleeves around the leads were removed (Figure 2c). The surgical field was thoroughly debrided of residual, potentially infected tissue. Haemostasis was achieved. The surgical field was thoroughly irrigated with TauroPace™. The accessible segments of the leads and the connector pins were wiped with swabs soaked with TauroPace™. The connector pins were plugged back into the ports on the old pulse generator. The torque wrench tip was dipped in TauroPace™ before insertion through the seals to tighten the set screws. A new sub-pectoral pocket was fashioned and irrigated with TauroPace™ (Figure 1d). The old pulse generator was positioned within the new sub-pectoral pocket (Figure 1e). The sub-pectoral pocket was “locked” with another 5cc of TauroPace^TM^ as it was closed. The skin edges were closed with interrupted sutures (Figure 2a). After application of the wound dressing, pressure was applied over the pocket by bandage under tension and a sandbag for 36 h. Mild local inflammation around the wound had disappeared by the second day.

The patient received a further 7 days of oral antibiotics to complete a 2-week course. The pacemaker wound looked healthy with no sign of recurrent infection, underlying abscess formation, or skin erosion 7 days after the procedure (Figure 2e).

Repeated laboratory testing for infection (full blood count, inflammatory markers, and blood cultures) remained negative. No clinical symptoms or signs of relapse of infection were observed at nine months follow-up in March 2022.

## 3. Discussion and Conclusions

The rate of CIED infection has increased significantly and in excess of the rise in implantation rate caused by expansion of therapy indications [1]. When a CIED is colonised by pathogens, total device system removal (including lead extraction) is the standard of care [3]. However, under rare circumstances, it would be preferable (if possible) or clinically necessary to make an attempt at salvaging the infected system to avoid its total removal [8,9]. Even if dormant sub-clinical bacterial colonisation remains, [21] as long as there is no overt clinical infection, this would be an acceptable clinical outcome.

The clinical success of the salvage procedure in this particular case could be attributed to several measures:

- the complete removal of all sutures and the lead anchor sleeves;
- the complete removal of all potentially infectious fibrous tissues encasing the pulse generator and accessible lead segments without contaminating the surrounding newly created (and theoretically sterile) operative field;
- stringent ex vivo sterilisation of the device hardware with TauroPace™, by total immersion where possible (the pulse generator, torque wrench tip) or vigorous external wiping with saturated swabs otherwise (lead segments, connector pins);
- stringent in vivo sterilisation of the operative field (the skin incision edges, the previous pre-pectoral pocket, the new sub-pectoral pocket) by thorough irrigation with TauroPace™;
- re-implantation in a “virgin” sub-pectoral position fashioned through a sterilised floor of the previous pre-pectoral pocket;
- “locking” the pocket with 5–10cc of TauroPace^TM^ before closing the wound;
- applying pressure over the device pocket to prevent haematoma formation.

Clinical guidelines provide general guidance on the management of common cases but should be interpreted with caution and applied with discretion in individual cases with exceptional circumstances. In frail patients with poor quality of life, significant comorbidities, or limited life expectancy, localised device infection may have to be managed conservatively—the operative risks of percutaneous lead extraction simply outweigh the potential benefits. This is currently most commonly achieved by frequent changing of wound dressings of the open device wound and/or chronic antibiotic therapy. Our case demonstrates the feasibility of achieving medium-term freedom from clinical infection with complete system internalisation and without total hardware removal of a primarily or secondarily infected pulse generator protruding through the skin in a frail patient. Compared to the disappointing results from previous attempts at infected CIED salvage [22,23], the clinical success in this case could be due to the novel use of TauroPace™ as an in vivo and ex vivo sterilisation agent of both the operative field and the system hardware [24,25].

This is the first report of TauroPace™’s use in the successful salvage of a partially exposed and polymicrobially infected CIED pulse generator. If an infected CIED can be successfully salvaged without complete removal and clinical relapse of infection, that would be immensely desirable. However, the “spectacular” success of the novel approach to CIED salvage procedure described in this report was followed up for only 9 months (currently constantly followed up). Therefore, whether it can be consistently replicated needs to be assessed in larger clinical studies.

## Data Availability

Please note that no data set was generated due to the nature of this publication.
